# Combined transcriptome and metabolite profiling analyses provide insights into the chronic toxicity of carbaryl and acetamiprid to *Apis mellifera* larvae

**DOI:** 10.1038/s41598-022-21403-0

**Published:** 2022-10-07

**Authors:** Jing Gao, Yang Yang, Shilong Ma, Feng Liu, Qiang Wang, Xing Wang, Yanyan Wu, Li Zhang, Yongjun Liu, Qingyun Diao, Pingli Dai

**Affiliations:** 1 Key Laboratory of Pollinating Insect Biology of Agriculture, Institute of Apicultural Research, Chinese Academy of Agricultural Sciences, Beijing, 100093 China; 2 Enshi Academy of Agricultural Sciences, Enshi, 445002 China; 3 Jiangxi Institute of Apicultural Research, Nanchang, 330201 China; 4 Beijing Apicultural Station, Beijing, 100029 China

**Keywords:** Entomology, Environmental impact

## Abstract

Despite many studies have revealed that developing honey bee (*Apis mellifera*) larvae are posting a high risk on exposure to insecticides, the toxicology information on bee larvae remain limited. The present study demonstrated the first assessment of the effects of no observed adverse effect concentration (NOAEC) of carbaryl (CR) and acetamiprid (ACE) on transcriptome and metabolome in honeybee larvae reared in vitro. Chronic exposure to carbaryl caused transcriptional disorders associated with oxidative stress. In addition, a series of metabolic homeostasis were disrupted by carbaryl stress, such amino acid metabolism, purine and pyrimidine metabolism and flavone and flavonol biosynthesis. The activities of enzymic biomarkers including GST, P450, CAT, AChE and SOD were not influenced by ACE stress, while the CR exposure slightly decreased the activity of CAT and SOD. Our results clearly show that ACE and CR display different potential to modulate transcriptome and metabolome associated with their different toxicity against bee larvae.

## Introduction

With the widespread application of synthetic organic pesticides in farmland or semi-forest land, the impairment caused by sublethal exposure to pesticides on honeybees at both individual and colony levels are widely recognized. It has become evident that constant exposure to sublethal doses of insecticides may have profound long-term effects on the dynamics of the entire colony, highlighting the imperative role of chronic toxic stress in colony decline^1–3^.

While traditional hazard assessment addressing effects of insecticides to honey bees before market only consider the lethal level (LD_50_) on adult worker bees after short-term exposure, this kind of poisonous assessment cannot accurately define the risks to honey bees or provide protective indicators. Although many countries have improved the assessment procedures of pesticide-related risks to non-target pollinator, uncertainties still exist regarding the regarding the data on exposure of pesticides that can be considered safe for bees^4^. As chronic effects have not been copiously characterized and standardized, the assessment of their consequences is more difficult and complicated^5–7^. A series of laboratory and field trials have suggested that the concentrations of insecticides contacted to or ingested by bees are usually far below known lethal level, but they may be at doses that cause negative effects on honey bees, such as learning, climbing and foraging behavior^1,8–10^. During the past few years, variable changes in gene expression and metabolites accumulation with chronic pesticide exposure have been observed in many laboratory-based studies. A recent study uncovered that exposure to sublethal dose of glyphosate disrupted the transcriptional and metabolic regulatory networks in honeybees, which may explain the negative effects on olfactory associative functions observed in exposed bees^11^. Notably, genes expression and metabolites abundance involved in glycolysis, lipids metabolism, and detoxification and immune systems were generally altered by treatments of sublethal dose of insecticides in honey bees^11–13^. These results highlight the molecular basis of the interactions between sublethal doses of pesticides and honey bees. Therefore, multiple tests should be required to evaluate the delayed sublethal effects of insecticides on honey bee colonies before authorization process, not only at the physiological level, but also at molecular level.

Dietary exposure through consumption contaminated pollen and nectar is the most important exposure route for adult and larvae of honey bees^14^. Thanks to continuously gathering contaminated pollen and nectar by foragers during the flowering period and transfer to hive, larvae in brood combs are usually chronically exposed to these compounds during the whole brood stage, especially considering that early life stages may be more sensitive to certain contaminants than adult stages. So far, more than 120 insecticides and their metabolites were identified in pollen and wax, most of which were at levels lower that the concentrations concentration expected to cause sublethal effects to bees^15,16^. The information reminds us that developing bee larvae present a high risk of exposure to insecticides, but information about the effects on oral toxicity to honey bee larvae is rare. Several studies have demonstrated that chronic oral exposure of larvae to insecticides significantly reduced larval survival, developmental rate and larval weight^17–19^. Particularly, synergistic effects may occur when pesticide exposure interact with pathogens or parasites of *A. mellifera*^20–22^. For instance, the combination of sublethal doses of thiacloprid and black queen cell virus (BQCV) significantly aggravated the proliferation of virus on host larval and potentially enhance the deleterious effects of the virus on honey bees^20^.

Systemic carbamates and neonicotinoids insecticides are commonly used in agriculture and have been reported to be toxic effects in honey bee larvae when consumed orally^23,24^. Both neonicotinoids and carbamates act on the cholinergic system of insect pests but with different mode of action. Neonicotinoids act as agonists of nicotinic acetylcholine receptors (nAChRs), causing paralysis and mortality of target pests by interfering with receptor signal transduction^25^. Carbamates kill insects by reversibly inactivating the enzyme activity of acetylcholinesterase^26^. Because of the potential effects on mammal health and environment, carbaryl was prohibited in the EU from 2007. Our previous study conducted a preliminary risk assessment of the chronic toxicity to *Apis* m*ellifer*a larvae of the representative products of these two insecticides, carbaryl and acetamiprid^27^. We found that the no observed adverse effect concentration (NOAEC) (https://www.epa.gov/sites/production/files/2015-11/beerexv1.0.xlsx) of the chronic toxicity test of acetamiprid (2 mg /L) and carbaryl (5 mg /L) to *A. mellifera* larvae were much higher than the field-realistic levels as well as the residual levels detected in bee products^28^. The risk of carbaryl and acetamiprid to bee larvae was lower than another two most common pyrethroid insecticides, cypermethrin and deltamethrin^27^. However, whether chronic exposure to carbaryl and acetamiprid could induce molecular change in honey bee larvae, i.e. how metabolites and gene expression of bees are affected by exposure of carbaryl and acetamiprid, typically remains unknown.

The purpose of this paper is to expand the risk assessment to the chronic effects of carbaryl and acetamiprid on the transcriptional and metabolic level of *A. mellifera* larvae at the concentration where no adverse reactions were observed. The network assessment of changed genes and metabolites could contribute to a better understanding of major molecular changes happening during pesticide exposure in bee developing stage.

## Results

### Identification of differentially expressed genes in *A. mellifera* larvae in response to carbaryl and acetamiprid

The sequencing assembly information is summarized in Table S1. About 7.04–9.03 Gb sequencing data of raw reads were obtained from each cDNA libraries. 331,979,711 clean reads ranging from 23,591,368 to 28,153,096 for each library were obtained. The Q30 percentage of the eight samples was over 95% for each library with an average GC content of 39.36%. In total, 10,959 unigenes were successfully matched either to a single or multiple genomic location. A Pearson correlation analysis showed that the R^2^ coefficients between each group of two biological replicates were greater than 0.918 (Fig. S1). Over 79.09% of the genes were generally expressed in all the treatments, representing 69% of the known honey bee genes (Fig. S2)^29^. These results indicate that the quality RNA sequencing profile of each biological replicate was high enough to be used to identify the differentially expressed genes (DEGs).

**Figure 1:**
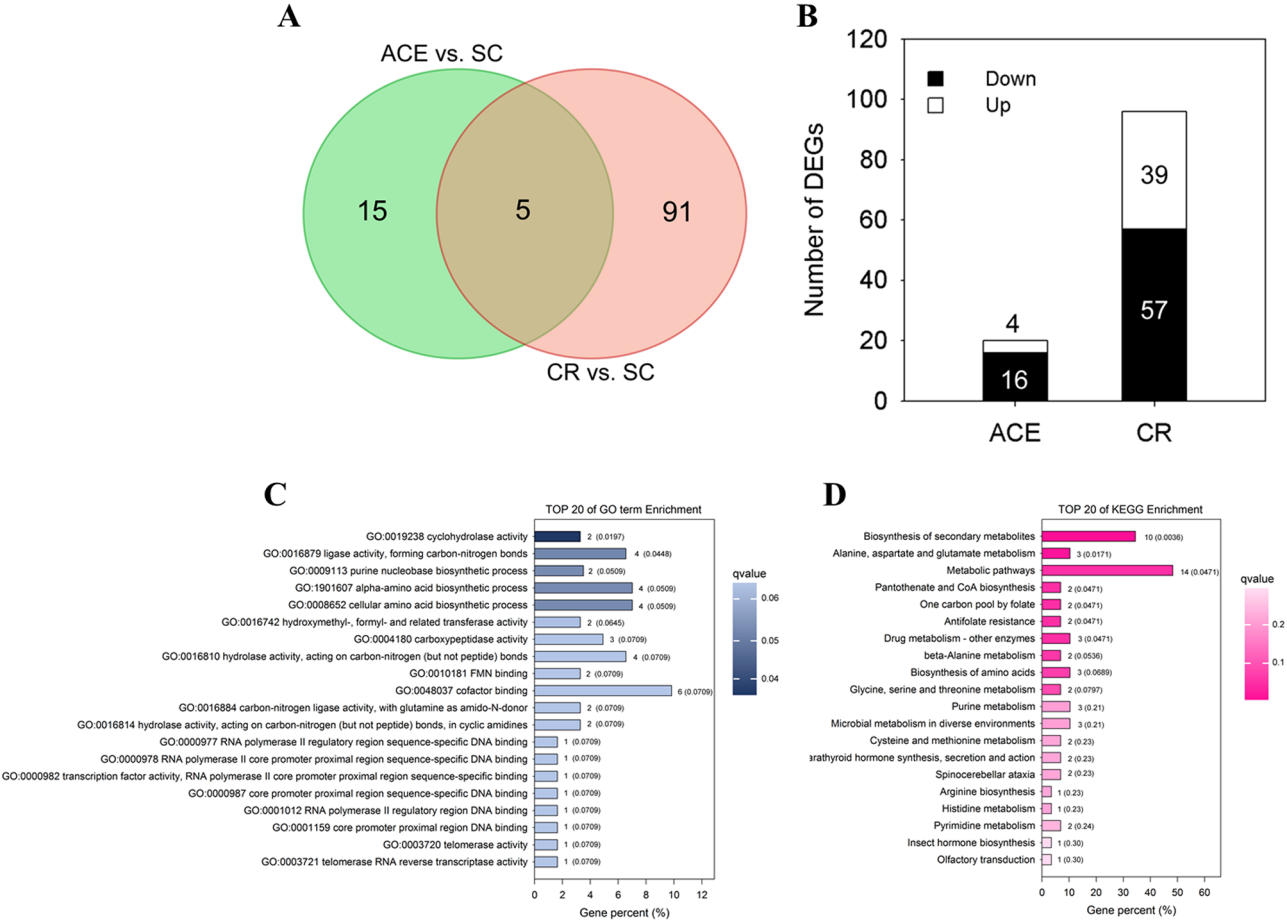
The differentially expressed genes (DEGs) between honey bees exposed to acetamiprid (ACE) or carbaryl (CR). (**A**) Venn diagram DEGs identified in honey bees exposed to ACE or CR. (**B**) Histogram of the number of DEGs identified in honey bees exposed to ACE or CR. (**C**) Go terms enrichment analysis of DEGs in *A. mellifera* larvae exposed to CR. (**D**) KEGG pathway term enrichment analysis of DEGs in *A. mellifera* larvae exposed to CR. The X-axis is the percentage of the differentially expressed genes in the total genes in the GO term or KEGG pathway. The number of genes and Q-values belonging to each Go category or KEGG pathway are shown behind the bars. Q value < 0.05 were considered significantly. The darker the color of the bar, the smaller the Q value, and the more significant the degree of enrichment.

To identify differentially expressed genes (DEGs) in the comparison between pesticide treated group and control group, the level of annotated *A. melifera* genes was calculated based on the basis of fragments per kilo base of exon per million fragments mapped (FPKM) metric. As very few differentially expressed genes identified in the comparison of NC (negative control) vs. SC (solvent control) (Table S2), solvent group (SC) was selected as representative negative group to make the following analysis more concise. Compared to the SC group, we found that 96 and 20 DEGs were differentially expressed in CR (carbaryl-treated) group and ACE (acetamiprid-treated) group, respectively (Tables S3 and S4). Venn diagram analysis revealed that 5 genes overlapped between the two comparisons, including oxidase 1 (GB46737), glycine *N*-methyltransferase (GB44871), nuclear receptor-binding factor 2-like (GB50147), asparagine synthetase (GB45340), and protein lethal(2)essential for life-like gene (GB45906) (Fig. 1A). Notably, the trends of these commonly differentially expressed genes were similar in both comparisons. Compared to SC group, there are 39 up-regulated genes and 57 down-regulated genes in carbaryl exposure treatment bees, while 4 up-regulated DEGs and 16 down-regulated DEGs were identified in the comparison of ACE vs. SC, (Fig. 1B). The very few differentially expressed genes observed in the comparison of ACE vs. SC implied that larvae exposed to NOAEC of acetamiprid have similarities in gene expression patterns as control group.

### Functional annotation and classification of carbaryl-responding genes

As only a small number of genes and only few with annotation information identified in ACE treated bees, enrichment analysis was not possible for functional analysis of acetamiprid-responding genes. Here, we will only focus on the assignment of biological functions to carbaryl-responding genes. Gene Ontology (GO) analysis indicated that 85 of the DEGs responding to carbaryl treatment had at least one matched into one of three main GO categories (biological process, molecular function and cellular component). As shown in Fig. 1C, the most enriched GO terms are “cyclohydrolase activity” (GO: 0019238) and “ligase activity, forming carbon–nitrogen bonds” (GO: 0016879) with Q value < 0.05, followed with “purine nucleobase biosynthetic process” (GO: 0009113), “α-amino acid biosynthetic process” (GO: 1901607) and “cellular amino acid biosynthetic process” (GO: 0008652), albeit not statistically significantly (Q value = 0.0509) (Table S5). Further, KEGG (Kyoto Encyclopedia of Genes and Genomes) pathway enrichment analysis was also conducted with the genes responding to carbaryl treatment. The top 20 KEGG pathways with the highest representation of DEGs are shown in Fig. 1D. Among these, 7 pathways were significantly enriched with a Q value of < 0.05 (Table S6), including “Biosynthesis of secondary metabolites” (ko01110), “Alanine, aspartate and glutamate metabolism” (ko00250), “Metabolic pathways”(ko01100), “One carbon pool by folate” (ko00670), “Drug metabolism—other enzymes” (ko00983), “Antifolate resistance” (ko01523), “Pantothenate and CoA biosynthesis” (ko00770). Particularly, genes that have been shown to involve in oxidative stress or redox homeostasis including hydroxyacid oxidase 1 (Hao1, GB46737), pyridoxine/pyridoxamine 5′-phosphate oxidase (PPOX, GB54776), methionine sulfoxide reductase A (MsrA, GB55004) and UDP-glucuronosyltransferase 1-3-like (UGT1A3, GB54485) were significantly decreased after treatment with carbaryl (Table S4).

### Metabolic profiles identified in *A. mellifera* larvae exposed to carbaryl and acetamiprid

**Figure 2:**
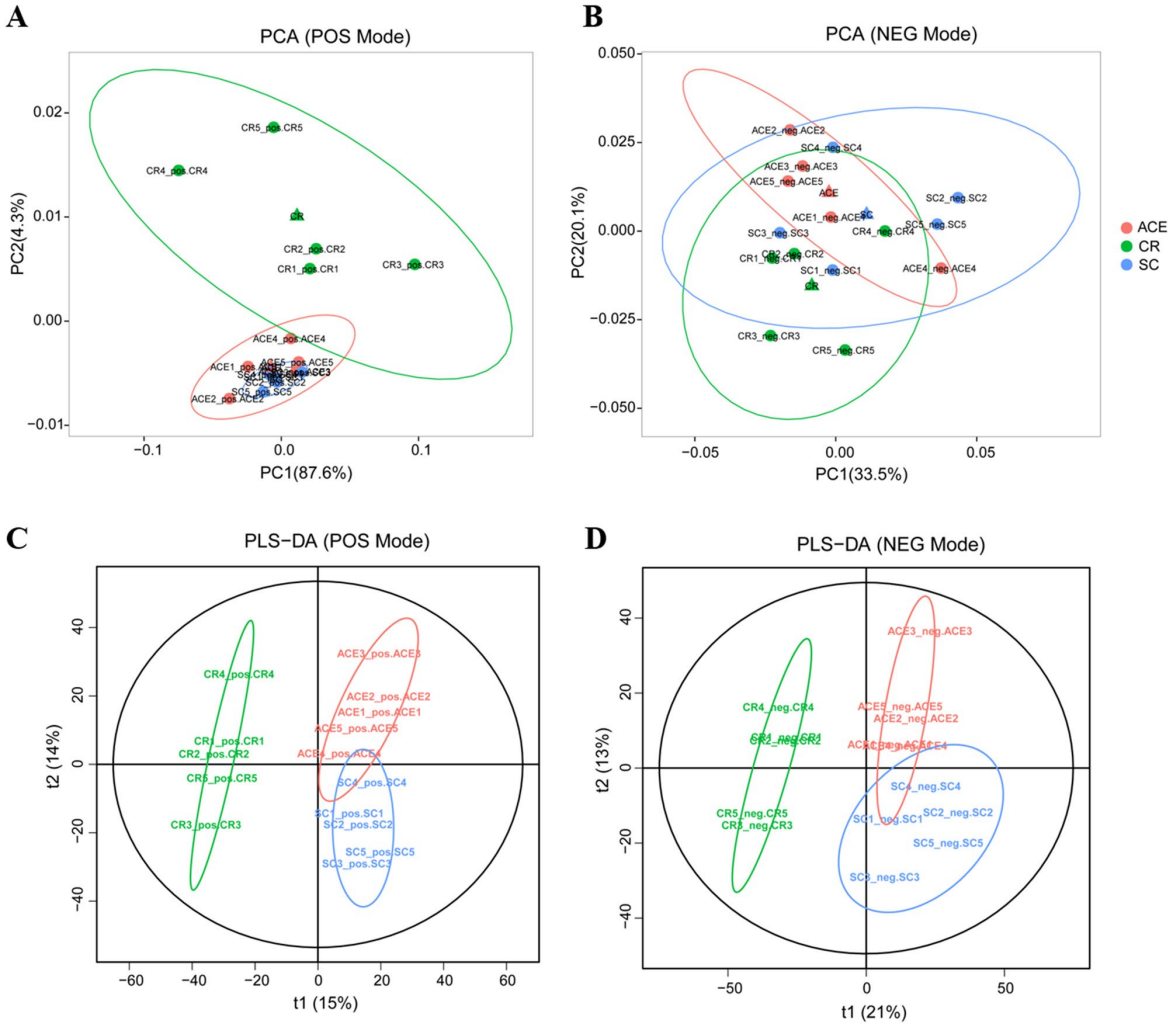
Principal component analysis (PCA) and Partial least-squares-discriminant analyses (PLS-DA) score scatter plot of the metabolite profiles identified in bees exposed to acetamiprid (ACE), carbaryl (CR) and solvent control (SC) in POS (**A**, **C**) and NEG (**B**, **D**) ion modes. (**A**, **B**) Principal component (PC) 1 and PC 2 represent the first two principal components accounting for a total of 91.9% and 53.6% of the variance under the positive and negative ion mode conditions, respectively; (**C**, **D**) t[1] = first principal component. t[2] = second orthogonal component. In POS mode, the three significant components with R2X, R2Y and Q2 values was 0.544, 0.98 and 0.758, respectively. In NEG mode, the three significant components with R2X, R2Y and Q2 values was 0.346, 0.754 and 0.336, respectively. Red, green, and blue symbols represent ACE treated samples, carbaryl treated samplesa and solvent control, respectively. Each condition was analyzed by five biological replicates.

As metabolite levels are the ultimate outcome of the response to environmental and genetic changes, we further evaluated the metabolic changes induced by the same treatment of carbaryl and acetamiprid in *A. mellifera* larval. After being exposed to 2 mg/L carbaryl or 5 mg/L acetamiprid for 4 days, the larvae were collected for analysis of metabolic changes under pesticide treatments. After removing low-quality ions, 3449 and 3694 ion peaks were screened in the NEG and POS modes, respectively. To visualize the metabolic changes in response to different pesticide exposure, these ions were processed by principal component analysis (PCA) and partial least squares discriminant analysis (PLS-DA) to predict the way the samples were grouped and distributed (Fig. 2). The non-supervised PCA scatter plot showed that the samples were clustered with respect to their groups (Fig. 2A,B). The first two principal components (PC1 and PC2) accounting for a total of 91.9% and 53.6% of the variance in POS and NEG modes, respectively. Next, the score plots of PLS-DA revealed a clear separation between the carbaryl treated group (CR) from acetamiprid treated group (ACE) and the solvent control (SC) in both ion modes (Fig. 2C,D). The results indicated that the significant changes in metabolites between the CR group and SC group. The metabolites in ACE group were indistinguishable from SC group, suggesting that the bee larvae do not respond strongly to acetamiprid stress at the metabolic level.

### Metabolic alteration in response to carbaryl and acetamiprid in *A. mellifera* larvae

**Figure 3:**
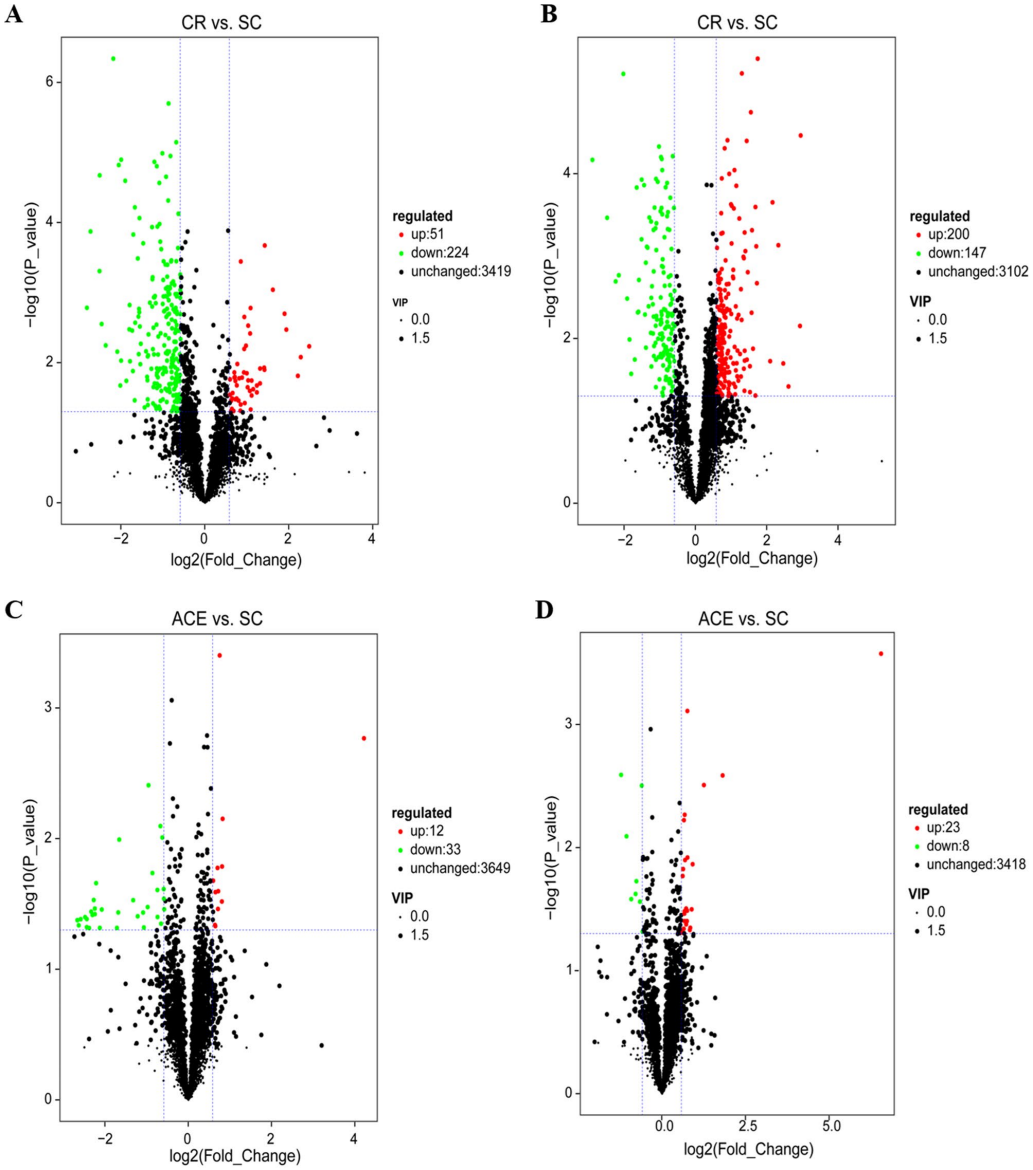
The Differentially expressed metabolites (DEMs) of *A. mellifera* larvae treated with carbaryl (CR) (Fig. 2A,B) or acetamiprid (ACE) (Fig. 2C,D) in this study. (**A**, **C**) Volcano plot for the positive ion (POS) mode; (**B**, **D**) Volcano plot for the negative ion (NEG) mode. Each point represents a metabolite, and the greater the scattered point, the greater the value of variable importance is in the projection (VIP). Up-regulated and down-regulated means that these metabolites were higher or lower expressed in treatment group compared to solvent control (SC).

**Figure 4:**
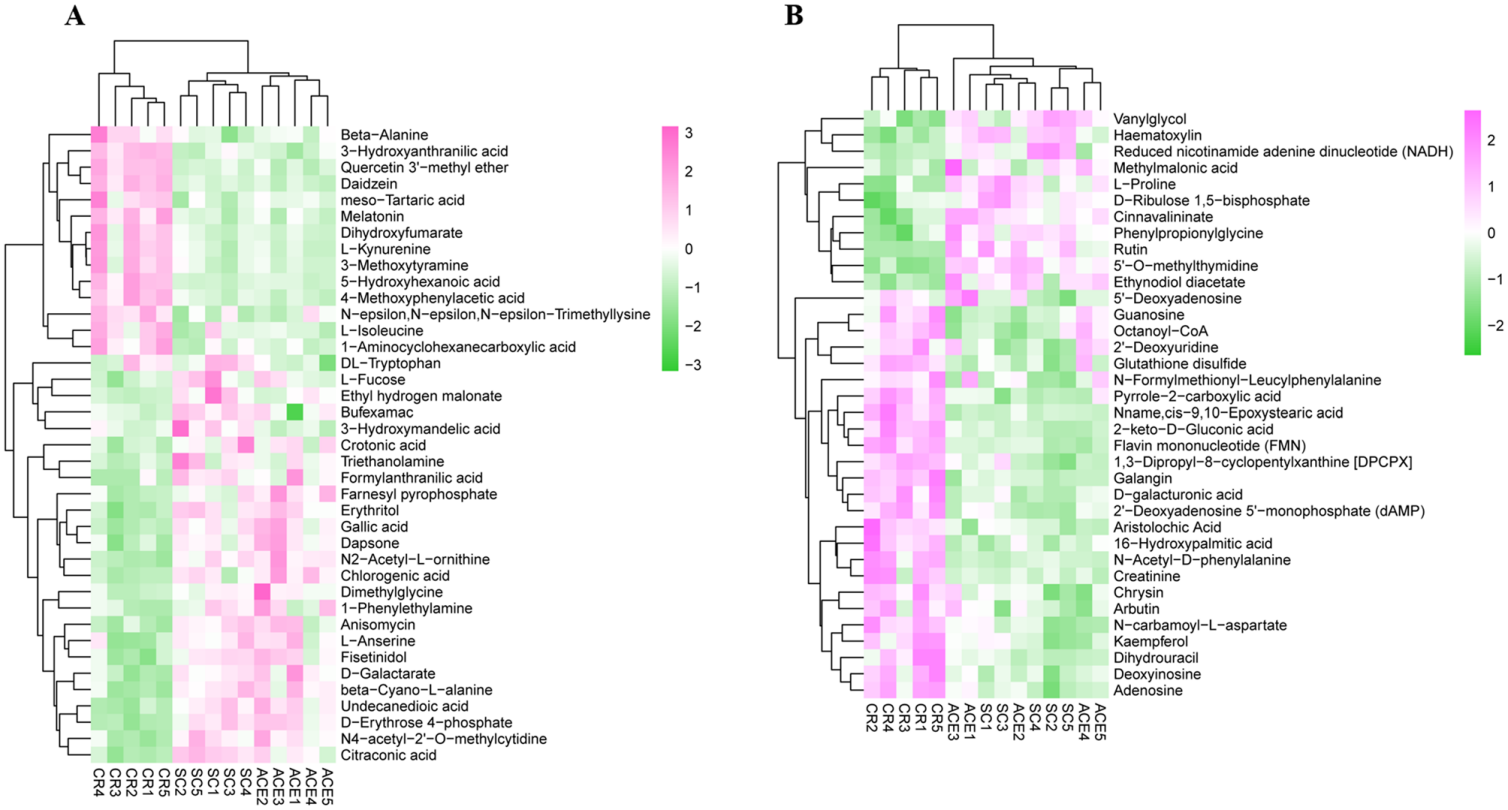
Hierarchical cluster analysis (HCA) and heat map of metabolite profiles of DEMs identified in comparison of carbaryl (CR) or acetamiprid (ACE) vs control bees (SC) in POS (**A**) and NEG (**B**) ion modes. HCA was performed using Z-score of the normalized quantitative values of the differential metabolites. The analysis was based on known metabolites selected at P < 0.05 of Student’s t-tests, VIP-values > 1.0 and │fold change│ ≥ 1.5 (Supplemental Data Sheets S5). The abscissa represents the independent replicates of different experimental groups, and the ordinate represents the selected metabolites. The color patches represent the relative expression of metabolites in the corresponding positions.

Statistical testing of pairwise comparisons with all tests set to a threshold of variable importance in projection (VIP) > 1, P < 0.05 (student's t-test) and fold change ≥ 1.5 to acquire differentially expressed metabolites (DEMs) responding to pesticide treatment. Volcano plots were used to represent the the significant difference between CR vs. SC and ACE vs. SC (Fig. 3). In the comparison of CR vs. SC, a total of 622 differential metabolites were identified, among which 347 (up 200, down 147) and 275 (up 51, down 224) metabolites were in the POS ion mode and NEG ion mode, respectively (Table S7). In addition, we obtained 31 (up 23, down 8) and 45 (up 12, down 33) DEMs in the comparison of ACE vs. SC in the POS ion mode and NEG ion mode, respectively (Table S8). We subsequently selected the subset of the relevant and known metabolites for hierarchical cluster analysis (HCA) applying Pearson’s correlation to classify metabolites with the same characteristics (Fig. 4). A total of 75 differential metabolites have been selected, including 39 metabolites detected in the POS mode and 36 metabolites detected in the NEG mode (Table S9). This set only included 4 annotated metabolites in the comparison of ACE vs. SC, such as DL-tryptophan (0.59-fold), 3-hydroxymandelic acid (0.50-fold), meclofenamate (0.66-fold), and 5'-deoxyadenosine (1.58-fold). In the POS mode, more than half of the differential metabolites in the CR treated group were down-regulated, while the trend in the NEG mode was opposite. Among the down-regulated metabolites, citraconic acid was the most down-regulated metabolite (0.24-fold), followed by formyl anthranilic acid (0.34-fold), reduced nicotinamide adenine dinucleotide (NADH) (0.38-fold) and ethyl hydrogen malonate (0.39-fold). Dihydrouracil (5.45-fold), melatonin (3.81-fold), and 5-hydroxyhexanoic acid (3.70-fold) were the top three up-regulated metabolites. Additionally, 7 metabolites related to Purine or Pyrimidine metabolism were up-regulated in the CR treated group (Table S10).

### Functional annotation and KEGG pathway enrichment analysis of DEMs in responding to carbaryl

**Figure 5:**
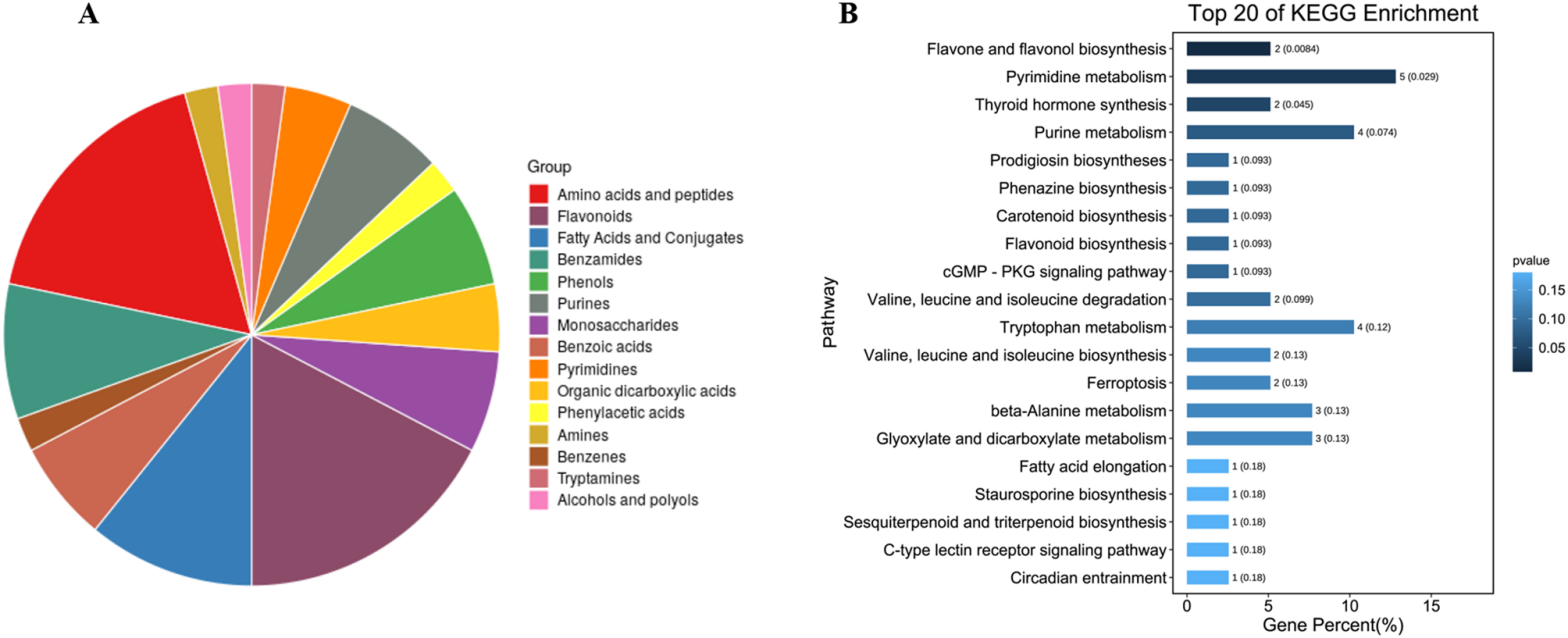
Exposure to carbaryl (CR) treatment changed the accumulation of metabolites involved in important pathways in larvae. (**A**) Component analysis of the DEMs in *A. mellifera* larvae exposed to CR. (**B**) KEGG pathway enrichment analysis of DEMs in *A. mellifera* larvae exposed to CR. The represent the number of differentially enriched metabolites, the y-axis represents the enrichment pathway. The darker the color of the bar, the smaller the P value, and the more significant the degree of enrichment.

We further focused on the function of DEMs in bee larvae subjected to carbaryl treatment. Among the DEMs responding to carbaryl exposure, amino acids and peptides (17.4%), flavonoids (17.4%), fatty acids and conjugates (10.9%), benzamides and (8.7%) accounted for a large proportion in CR treated group compared to solvent control (Fig. 5A). Most amino acid including l-proline, phenylpropionylglycine, *N*2-acetyl-l-ornithine, dimethylglycine, l-anserine and dl-tryptophan, were significantly decreased in bee larvae after exposure to carbaryl. In contrast, l-isoleucine, β-alanine and *N*-carbamoyl-l-aspartate were significantly increased under carbaryl stress (Table S9). Additionally, we observed that the contents of most nucleic acids (including 2′-deoxyadenosine 5′-monophosphate, flavin mononucleotide, 2′-deoxyuridine, deoxyinosine, adenosine, and dihydrouracil) were significant higher in carbaryl treated larvae. To identify the metabolic pathways disturbed by carbaryl exposure, the DEMs were subjected to KEGG pathway enrichment analysis. The top 20 KEGG pathways with the representation of DEMs are shown in Fig. 5B and Table S10. We found that the terms “Flavone and flavonol biosynthesis”, “Pyrimidine metabolism”, and “Thyroid hormone synthesis” were significantly enriched (*p* > 0.05).

### Correlation analysis of the DEGs and DEMs in *A. mellifera* larvae exposed to carbaryl

**Figure 6:**
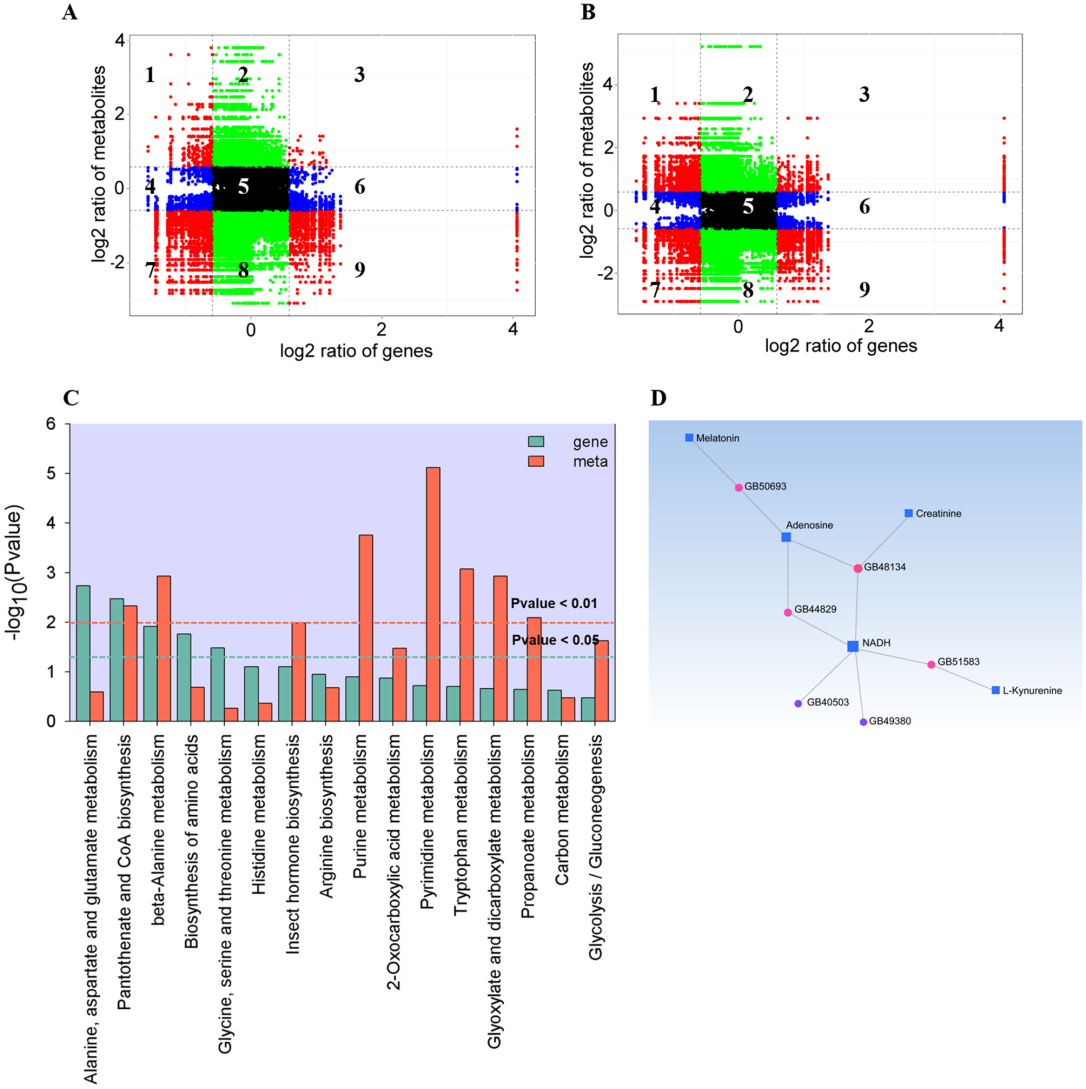
Correlation analysis of the transcriptomic and metabolomic data of *A. mellifera* larvae exposed to carbaryl. (**A**, **B**) Quadrant diagrams representing the association between the gene identified in RNA-seq analysis and metabolites identified with UHPLC-MS/MS in POS (**A**) and NEG (**B**) mode. The x-axis represents that the log ratio of gene and the y-axis represents the log2 ratio of metabolite; each dots represents individual gene or metabolite; black dotted lines represent the different threshold; black dots represent the unchanged genes or metabolites; green dots represent DEMs with unchanged genes; bule dots represent DEGs with unchanged metabolites; red dots represent both DEGs and DEMs. (**C**) KEGG enrichment analysis of the DEGs and DEMs that were enriched in the same pathway. (**D**) Interaction network depicted multiple hubs of gene-metabolite interaction under carbaryl stress. The network was generated based on the potential interaction data from the (MetaboAnalyst 5.0). Circle nodes represent genes, and square noted represent metabolites. Node size indicates its connectivity measured as node degree (i.e., the number of edges connecting the node); the bigger node means a higher connectivity. The edge indicates the interaction between gene and metabolites and the weight of edges between them is a measure of their interaction. All interaction data are available in Supplementary Table S12 in “Supplementary Material”.

We also analyzed the correlation between metabolome and transcriptome in *A. mellifera* larvae exposed to carbaryl in the present study. The variations of the metabolites and their associated genes with the Pearson correlation coefficient over 0.8 were selected to draw nine quadrant diagrams. As shown in Fig. 6A,B, the diagram divided genes and metabolites into nine quadrants based on their different expression pattern (Table S10). The quadrant 7 and quadrant 9 had a higher number of dots, suggested that more DEMs and DEGs were positively correlated in the quadrant 7 and negatively correlated in the quadrant 9. Dots accumulated in Quadrant 7 and quadrant 3 represent expression patterns of genes and metabolites consistent with each other. More dots accumulated in quadrant 7 suggested that more down-regulated DEMs and DEGs than up-regulated DEMs and DEGs were positive correlated (Fig. 6A,B).

To further evaluate the effects of the transcriptome variation on the metabolite accumulation in *A. mellifera* larvae, the DEMs and DEGs responding to carbaryl treatment were submit to the KEGG pathways^30^. We found that a total of sixteen pathways contained both DEGs and DEMs, among which “Alanine, aspartate and glutamate metabolism (ko00250)”, “Purine metabolism (ko00230)”, “Pantothenate and CoA biosynthesis (ko00770)”, “Tryptophan metabolism (ko00380)”, “Pyrimidine metabolism (ko00240)”, “β-Alanine metabolism (ko00410)” were found to be the most relevant (Fig. 6C). For instance, *N*-carbamoyl-l-aspartate showed a strong positive correlation with a gene encoding amidophosphoribosyltransferase (GB52260) (R^2^ = 0.96) and they were enriched in the same pathway related to Alanine, aspartate and glutamate metabolism. A gene encoding kynurenine/α-aminoadipate aminotransferase (GB51583) and cinnavalininate was negative correlated (R^2^ = − 0.81) and enriched in the same “Tryptophan metabolism pathway” (ko00380) (Table S12). These results implied that the DEGs may play a role in the alteration of the corresponding metabolites via direct or indirect regulation. Further, we constructed a gene to metabolite network with the data drawn from metabolomics and transcriptomics analysis to uncover a comprehensive gene regulatory mechanism that involves carbaryl stress. As shown in Fig. 6D, 5 genes and 6 metabolites were linked to the network, in which reduced nicotimide adenine dinucleotide (NADH) acted as the core component (Table S13). Taken together, these results indicate that CR exposure affects the transcriptomic and metabolomic regulatory networks in honey bees.

### Influences on the activities of enzymatic biomarkers

**Figure 7:**
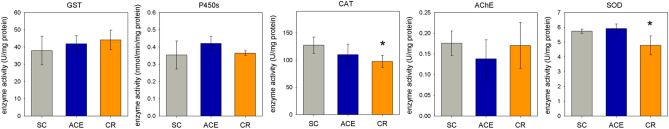
Effects of ACE and CR on enzymes activity of GST, P450s, CAT, AChE and SOD in bee larvae after exposure. Data are means of five biological replicates, and error bars represent ± standard error (SE) (n = 5).* P* < 0.05 are indicated by an asterisk above the bars and were determined by one-way ANOVA followed by Bonferroni’s post hoc test.

Enzymes that protect honey bees in pesticide-loaded environments are considered as sensitive biomarkers, especially those involved in and detoxification processes and oxidative stress. Although no mortality or other obvious negative physiological effects could be observed, the alternation of biomarkers could be regarded as a spectator of physiological changes induced by the pesticides in developing stage. Therefore, the activities of enzymes that were commonly considered as enzymatic biomarkers of exposure to xenobiotics in the honey bee were also assessed. As shown in Fig. 7, the activities (U/mg proteins) of glutathione-S-transferase (GST), cytochrome P450 monooxygenases (P450) and acetylcholinesterase (AChE) were not influenced by carbaryl and acetamiprid, while the carbaryl slightly decreased catalase (CAT) activity (− 23.4% of control, P < 0.05) and superoxide dismutase (SOD) activity (− 16.6% of control, P < 0.05).

## Discussion

In current agricultural practices, honey bees are getting increasingly risk of exposure to insecticides as a consequence of foraging behavior in farmland^31^. Many scientific studies are overly inclined to report on negative issues, no matter how weak the findings that suggest problems, rather than seek to the balance of bee health and pesticide application. The concentration used in some of the recent laboratory studies were more representative of “worst case” than the “field realistic” dose^32–34^. It has been suggested that the transcriptional alternation induced by pesticides was generally with a concentration-dependent effect^12^. Thus, in order to avoid overestimating the negative effects of pesticides on insecticides, the dose chosen is critical. In our previous study, we measured the no observed adverse effect concentration (NOAEC) of carbaryl (CR) and acetamiprid (ACE) on developing larvae, which were 48 times and 376 times the maximum residual value in nectar or honey, respectively^15,28^. We found that the risk of chronic exposure of these insecticides at NOAEC to bee larvae were acceptable at a physiological level^27^. The objective of this study was to further test whether honey bee larvae were adversely affected in terms of transcriptome and metabolome when exposed to carbaryl and acetamiprid at the level where no adverse effects were observed. Our findings suggested that carbaryl and acetamiprid exhibited different potentials in modulating transcriptome and metabolome, consistent with their toxicity against *A. mellifera* larvae^27^.

Acetamiprid is a representative of the first generation of neonicotinoids with broad-spectrum characteristic. Due to its low acute toxicity on bees, acetamiprid is currently the primarily available neonicotinoid pesticides in EU^35^. Our transcriptional profiling data showed that very few differentially expressed genes could be identified in larval exposed to NOAEC of acetamiprid (ACE), at least using the statistical methods (DESeq 2) in the present study. Of the 20 DEGs identified in the ACE-treated group, five genes were also found to be differentially expressed in the carbaryl (CR) treatment, including glycine N-methyltransferase-like (GB44871) and asparagine synthetase (GB45340), hydroxyacid oxidase 1 (GB46737), nuclear receptor-binding factor 2-like (GB50147), and protein lethal(2)essential for life-like gene (GB45906) (Fig. 1A). Glycine N- methyltransferase-like (GNMT) has been found to maintained high level throughout larval development in the queen caste^36^. In mammal, the homeostasis of GNMT expression is very important for the cellular defense against exogenous stress. GNMT catalyzes the synthesis of sarcosine from glycine using S-adenosylmethionine (SAM) and regulates genes related to detoxification and anti-oxidation pathways^37^. In the present study, GNMT were both down-regulated in ACE treated and CR treated group, which may result in decreased ability of detoxification and metabolization of xenobiotic compounds in exposed developing bee larvae eliminating the xenobiotic compounds. A previous research has indicated that sublethal acetamiprid doses had a negative effect on the cognitive ability of honey bees^38^. It has been suggested that asparagine synthetase was down-regulated after appetitive olfactory conditioning in honey bees^39^. The significant reduction of asparagine synthetase in acetamiprid or carbaryl exposed bee would directly influence the homeostasis of the predominant excitatory neurotransmitters glutamate and aspartate and potentially in turn impair memory acquisition. Chaperone/heat-shock proteins are involved in the protein-folding quality control machinery, in maintaining cellular protein homeostasis to adapt to the unnatural environment. Protein lethal (2) essential for life-like (GB45906) is a small heat shock protein that has been found commonly up-regulated in virus-infected bees^40–43^. The significant down-regulation of protein lethal (2) essential for life-like in exposed larval implied that sublethal dose of acetamiprid and carbaryl could depress the heat shock response and proteostasis network, thereby increasing pathogen loads and infection. According to the metabolomics profile in this study, the accumulation of metabolites in ACE treated group were indistinguishable from solvent control group (Figs. 2 and 4). The results were consistent with the transcriptome analysis that few genes involved in metabolism was identified in the comparison of ACE vs. SC.

Additionally, both mRNA expression levels and enzyme activity of GSTs, CaE, P450s, AChE and SOD did not change significantly in acetamiprid exposed larvae (Table S2 and Fig. 7). A previous study has shown that the enzyme activity of GST and CAT in adult honey bees were significantly stimulated by 0.01- and 0.02-fold of the field application dose of acetamiprid (0.6 and 1.2 mg/L, respectively), while the activity of GST and AChE were significantly decreased at higher application dose (2.4 and 6.0 mg/L)^44^. The modulation of enzyme activity of P450s, GST and CAT were also observed in *Apis cerana* bees after exposed to acetamiprid^45^. Shi et al. revealed that relative expression of *CYP450* and *Ace2* were up-regulated in the early life stage, while significantly down-regulated in adults after long-term exposure^46^. It is worthwhile to note that the dose of acetamiprid applied in this study (5 mg/L) did not differ significantly from the exposure dose of adults reported by other authors^44,46^. Whether the *A. mellifera* larvae are less sensitive to acetamiprid than adult is questionable and requires further investigation. Notable, the acetamiprid or its metabolites were not detected in the metabolic profiles (Table S7). It might be because the low dose of pesticide can be quickly distributed and detoxified in larval, thereby resulting in relatively low toxicity against honey bee^27,35,47^. Taken together, our data suggested that chronic exposure to NOAEC of acetamiprid had a very limited impact on worker larvae at molecular level (Figs. 2 and 4).

The comparisons of greatest interest in the present study are those for carbaryl (CR) treated larvae. CR is one of the broad-spectrum carbamate insecticides, and its primary mechanism involved the inhibition of acetylcholinesterase activity of the target insects^48^. Chronic exposure to CR has several lines of negative effects on adult bees, e.g., nesting performance, foraging ability and gut microbial community^49–51^. A previous study have demonstrated that sublethal doses of carbaryl altered the expression of immune and antioxidant related genes in worker bees, which may cause a physiological cost to honey bee healthy^52^. In this study, we found that CR disrupted the transcriptional and metabolic regulatory networks of bees, even though no adverse physiological effects were observed in exposed larvae. The transcriptome analysis revealed that 96 DEGs were identified between exposed larvae and control bees, and more DEGs were involved in nucleotide biosynthesis process, amino acid biosynthesis process, carbohydrate and energy metabolism, and oxidation–reduction process (Fig. 1C,D). In addition, metabolome analysis showed that CR treatment lead to reduction of amino acids, accumulation of nucleic acid components, and disturbed flavonoids and fatty acids in exposed larvae (Fig. 5). These results implied that chronic exposure to CR might change internal metabolism in bee larvae. To our knowledge, this is the first investigation of the molecular mechanism underlying *A. mellifera* responds to chronic toxicity of carbaryl.

Numerous studies have demonstrated that oxidative stress may be a common phenomenon caused by sublethal pesticide exposure in honey bees^53,54^. We found several genes associated with oxidative stress resistance or redox homeostasis were down-regulated in the CR exposed larvae. Methionine sulfoxide reductases (Msrs) is a critical enzyme in the conversion of methionine sulfoxide to methionine and participates in cell and tissue protection. The down-regulation of *MsrA* may enhance levels of methionine sulfoxide thereby lower the cellular antioxidant capacity in larvae. UDP-glucuronosyltransferases (UGTs), being major enzyme in the reaction of glycosylation, catalyze the detoxification of reactive metabolites thereby acting as indirect antioxidants^55,56^. We observed a UDP-glucuronosyltransferase (UGT1A3, GB54485) was significantly decreased after treatment with carbaryl (Table S4). A previous study reported that the expression of UGTs in adult Asian honeybees (*A. cerana cerana*) was highly induced by various pesticides^55^. In addition, silencing of a UDP-glucuronosyltransferase (AccUGT2B20) significantly reduced expression levels of antioxidant genes (e.g. SOD, POD, and CAT), suggesting potential detoxification properties of UGT that contribute to the regulation of downstream antioxidant genes^55^. In the present study, slightly decrease in CAT and SOD activity was also observed after larvae exposed to exposure, despite the transcriptional levels of the related enzymes were not significantly changed (Fig. 7). These results implied that both CAT and SOD help in resisting the oxidative stress induced by carbaryl pesticide, which supported by the findings in other species^57–59^. All of the above results revealed that the down-regulation of the genes for proteins involved in antioxidant defense as well as activity of CAT and SOD resulted in elevated levels of H_2_O_2_ in exposed larvae, and may further caused oxidative damage to proteins, nucleic acids, lipids and carbohydrates^60^. As there is often a weak correlation between RNA levels and the abundance of the corresponding proteins, it is difficult to establish a direct correlation between enzyme activity and mRNA expression level^61^. Thus, it is not surprising that the alterations in CAT and SOD activity reported herein were not associated to related gene expression. Whether the changes in CAT and SOD activity induced by CR are contributed by the altered protein abundance or other regulatory mechanisms such as post-translational modification is questionable and requires further investigation.

The pesticide-induced oxidative stress could disrupt a series of metabolic homeostasis, such as disorder metabolism of amino acid, nucleic acids and fatty acids^62^. We found that the responding genes and metabolites were most relevant to amino acid metabolism and purine and pyrimidine metabolism (Fig. 6C). These comparisons of genes and metabolites showed a major change in metabolism, with decreased amino acid metabolism and increased purine and pyrimidine metabolism. The contents of most changed amino acids were significantly decreased in bee larvae after exposure to carbaryl, such as l-proline and dl-Tryptophan (Table S7). The accumulation of proline can reduce stress-induced cellular acidification or primary oxidative respiration by removing reactive oxygen species in cells^63^. Tryptophan and its metabolites can improve cellular antioxidant capacity as they can efficiently clean free radicals^64,65^. In this study, one gene encoding kynurenine/α-aminoadipate aminotransferase (GB51583) and four metabolites (d-erythrose 4-phosphate, l-kynurenine, 3-hydroxyanthranilic acid and cinnamate) related to tryptophan metabolism were modulated by CR exposure (Fig. 6C and Table S10). Purine and pyrimidine metabolism produce DNA and RNA nucleotides and nucleosides and is involved in energy metabolism^66^. According to the results, reduced nicotimide adenine dinucleotide (NADH) and adenosine acted as the core component in the gene-metabolite network with the data drawn from metabolomics and transcriptomics analysis Fig. 6D. The upregulation of these purine and pyrimidine metabolites may implied that the exposed larvae reduce oxidative damage by accelerating cell metabolism. Altogether, our results suggest that carbaryl may affect the antioxidant capacity of honey bee larvae, and should contribute to understanding honeybee colony decline.

The transcriptomic and metabonomic approaches have been successfully applied to discover biomarkers associated with environmental stressors, toxicant response and disease diagnosis and monitoring^67,68^. Through a metabolomics-based approach, Wang et al. identified 8 significantly altered metabolites that were designated as candidate biomarkers for nutritional stress in *Bombus terrestris*^69^. Although a series of enzymatic biomarkers have been developed as appropriate biomarkers for risk assessment of exposure of the honeybee to pesticides, several genes or metabolites involved in immune system alteration, genotoxicity, olfactory signal transduction may also constitute potential biomarkers, as some of these have been observed in pesticide exposure studies^70,71^. Our data imply that genes and metabolites such as UGTs and tryptophan derivatives with drastic modulation involved in oxidative stress may be suitable biomarkers for evaluating the effects of xenobiotics on honeybees, and further exploration and strong research efforts are needed.

In conclusion, the present study demonstrated for the first time that carbaryl (CR) and acetamiprid (ACE) had distinct actions of transcriptional and metabolic regulation of honey bee larvae, which provide new insights into the chronic toxicity of carbamate and neonicotinoid against honey bee. We found that numbers of differentially expressed genes and metabolites in CR exposed bees were significantly higher than those in ACE exposed bees, consistent with their larval toxicity described in our previous study. The adverse effects of CR on internal metabolism in bee larvae at no observed adverse effect concentration should be given due consideration for its application in agricultural production.

## Materials and methods

### Insect samples

*Apis mellifera* worker larvae were obtained from five healthy hives at from an apiary in the Institute of Apicultural Research, Chinese Academy of Agricultural Sciences, in Beijing (N 39° 59′ 35.33″, E 116° 11′ 59.74″). The collection and feeding methods of honey bee larvae using the methodology described in our previous study^27^. For short, queens were caged onto an empty comb for 24 h to allow them to lay eggs. Then, successfully hatched larvae were collected and transferred to sterilized 48-well cell culture plates (STCPs) and reared in an incubator at 94% RH and 35 °C in the dark.

### Exposure to pesticides

Technical grade acetamiprid (purity 99.7%) and carbaryl (purity 98.9%) were purchased from Dr. Ehrenstorfer GmbH Corporation, Germany. Stock solution of acetamiprid or carbaryl was prepared by dissolving the powder in acetone and then diluted with diet C (50% royal jelly, 2% yeast extract, 9% d-glucose, 9% d-fructose) as described in Schmehl et al.^72^. The final concentration of 2 mg/L carbaryl or 5 mg/L acetamiprid were applied to the third instar larvae for 4 days. The concentrations respectively correspond to the NOAEC determined in our previous study^27^. The solvent solution for control groups was identical but pesticide free (The solvent accounted for 0.5% of the final diet). Samples were collected for enzyme activity assay or immediately frozen in liquid nitrogen and stored at − 80 °C until RNA and metabolites extraction.

### RNA isolation, library construction and RNA-Seq

Honey bees larvae treated as described above were used for transcriptomic analysis. Ten larvae from the same plates were pooled together as one replicate. For each experimental group, four or five biological replicates were isolated. The total RNA of each sample was isolated with Quick RNA isolation kit from Bioteke Corporation, China. The RNA integrity and quality were assessed by 1% agarose gel electrophoresis and Agilent 2100 Bioanalyzer (Agilent Technologies, Santa Clara, USA), respectively. The mRNA-Seq libraries were created using NEBNext^®^ Ultra™ RNA Library Prep Kit for Illumina^®^ (NEB, Ipswich, Massachusetts, USA) following manufacturer’s recommendations. The RNA-Seq were performed on an Illumina Hiseq 2000 platform (Illumina Inc., San Diego, CA, USA) following the standard Illumina preparation protocol by Biomarker Biotechnology Corporation (Beijing, China).

### Bioinformatics analysis of RNA-seq data

After removing the raw reads with adaptors of low quality (> 50%) or a high proportion of unknown bases (> 5%), the high quality reads were mapped to the *A. mellifera* genome (https://www.ncbi.nlm.nih.gov/assembly/GCF_000002195.4/) using the Trinity platform with a maximum allowance of 2 nucleotide mismatches and all other parameters set default^73^. The assembled unigenes were queried against in a series of public databases including NCBI-nonredundant protein (NR), Swiss-Prot protein database (Swiss-Prot), Clusters of Orthologous Groups (COG) to obtain their functional annotations^74^. The gene expression level was performed by the fragments per kilobase of transcript per million fragments mapped (FPKM)^75^. To test the reliability of RNA-seq data in the experiments, Pearson correlation index was calculated among the samples according to the gene expression based on the mean values. The closer the correlation coefficient R^2^ is to 1, the higher the similarity of expression patterns between two samples. If the R^2^ between two biological replicates is less than 0.8, the experiment needs to be re-run. Differentially expressed genes (DEGs) were implemented by the DESeq2 R package (1.18.0) with the threshold of │fold change│ ≥ 1.5 and a false discovery rate (FDR) ≤ 0.01. Successively, functional classification of the DEGs was implemented by the topGO R packages based on Kolmogorov–Smirnov test, and the statistical enrichment of DEGs in KEGG pathways were performed by KOBAS software against the KEGG (Kyoto Encyclopedia of Genes and Genomes) database (http://www.kegg.jp/).

### Metabolites profiling and data analysis

The larvae used for metabolome analysis were the same batch of samples used for the transcriptome. The metabolites extraction of the developing larvae was carried out as previously described^76^. The metabolite identification and quantification were performed using an UHPLC system (1290, Agilent Technologies) coupled to TripleTOF 5600 (Q-TOF, AB Sciex) at Shanghai Biotree biotech Co., Ltd. (http://biotree.cn/index_en) following their standard procedures and previously described by Yang et al.^77^. Metabolite profiles were determined in five control and five treatment groups with each sample consisting of 10 homogenised larvae. For a full description of sample preparation, mass spectrometry and data analysis, see “Supplementary Materials and Methods”.

### Correlation analysis of transcriptomic and metabolomic data

The correlation analysis of transcriptomic and metabolomic data was conducted by calculating the Pearson correlation coefficient (R) for the differentially expressed genes and metabolites with the cor function in the R package. The variations in the metabolites and their corresponding genes with the Pearson correlation coefficient over 0.8 were used to draw nine quadrant diagrams by using ggplot2 and getopt. A combination analyses was performed on the datasets from both metabolomics and transcriptomics using MetaboAnalyst 5.0 “Joint Pathway Analysis” and “Network” feature (http://www.metaboanalyst.ca)^30^.

### Enzyme activity assay

After exposure to pesticide, three larvae were pooled as one sample and were precooled in sterile deionized water containing 0.9% NaCl and were homogenized on ice. After centrifuged at 10,000*g* for 10 min, the supernatant fractions of the crude protein extract were used to measure enzymes activity. Protein concentrations were determined by Bradford micro-assay (Bio-Rad, www.bio-rad.com). The activity of glutathione-S-transferase (GST), catalase (CAT), acetylcholinesterase (AChE), and superoxide dismutase (SOD) in larvae was determined using commercial assay kits (Nanjing Jiancheng Bioengineering Institute, China) following the manufacturer's protocols. One unit of enzyme activity was defined as the amount of enzyme that degraded one unit of substrate supplied with the kits. The enzyme activities of all tested enzyme were represented with enzyme units (U) per protein content (U/mg protein). Activity of cytochrome P450 monooxygenases (P450s) was determined by method as described in Shimada et al.^78^. P450s activity was tested using 7-ethoxycoumarin as a substrate and expressed as nmol of 7-hydroxycoumarin formed per min per mg protein (nmol/min/mg protein). All enzyme activity tests were performed five times with independent biological replicates. The statistical differences between treatments and controls were tested with One-way analysis of variance (ANOVA) followed by Benjamini & Hochberg test using IBM SPSS Statistics 19 (IBM Corp., Armonk, NY, USA).

## Supplementary Information

Supplementary Information.

Supplementary Table S1.

Supplementary Table S2.

Supplementary Table S3.

Supplementary Table S4.

Supplementary Table S5.

Supplementary Table S6.

Supplementary Table S7.

Supplementary Table S8.

Supplementary Table S9.

Supplementary Table S10.

Supplementary Table S11.

Supplementary Table S12.

Supplementary Table S13.

## Data Availability

The data sets supporting the results of this article (Additional file 1: Tables S1–S12) are included within the article and its additional files. More details can be obtained from the corresponding author (daipingli@caas.cn) upon request.

## Abbreviations

GST: Glutathione-S-transferase
P450: Cytochrome P450 monooxygenases
CAT: Catalase
AChE: Acetylcholinesterase
SOD: Superoxide dismutase
BQCV: Black queen cell virus
NOAEC: No observed adverse effect concentration
DEGs: Differentially expressed genes
DEMs: Differentially expressed metabolites
PCA: Principal component analysis
PLS-DA: Partial least squares discriminant analysis
NADH: Reduced nicotinamide adenine dinucleotide
MsrA: Methionine sulphoxide reductase A
Hao1: Hydroxyacid oxidase 1
PPOX: Pyridoxine/pyridoxamine 5′-phosphate oxidase
UGT1A3: UDP-glucuronosyltransferase 1-3-like
