# Supplementary material for: Combined transcriptome and metabolite profiling analyses provide insights into the chronic toxicity of carbaryl and acetamiprid to Apis mellifera larvae
Source: Sci Rep. 2022 Oct 7;12:16898. doi: 10.1038/s41598-022-21403-0 (PMC9543932; doi:10.1038/s41598-022-21403-0)
Supplement: Supplementary file 1 — Supplementary Information. [file 41598_2022_21403_MOESM1_ESM.pdf]

SUPPLEMENTARY INFORMATION

Combined transcriptome and metabolite profiling analyses provide insights into the chronic toxicity of carbaryl and acetamiprid to *Apis mellifera* larvae

Jing Gao [a, #] Yang Yang,[a, #] Shilong Ma,[a,b] Feng Liu,[c] Qiang Wang,[a] Xing Wang,[d]

Yanyan Wu,[a] Li Zhang,[a] Yongjun Liu,[a] Qingyun Diao,[a] Pingli Dai[a*]

** Correspondence to: P Dai, Key Laboratory of Pollinating Insect Biology, Institute of Apicultural Research, Chinese Academy of Agricultural Sciences, Beijing 100093, China. E-mail: daipingli@caas.cn.*

a Key Laboratory of Pollinating Insect Biology of Agriculture, Institute of Apicultural Research, Chinese Academy of Agricultural Sciences, Beijing 100093, China

b Enshi Academy of Agricultural Sciences, Enshi 445002, China

c Jiangxi Institute of Apicultural Research, Nanchang 330201, China

d Beijing Apicultural Station, Beijing 100029, China

[#] *These authors have contributed equally to this study.*

Figure S1. A Pearson correlation analysis of repetitive samples in this study. (PDF)

Figure. S2. Venn diagram of unigenes identified in CR (exposure to carbaryl), ACE (exposure to acetamiprid) and SC (solution control) group. (PDF)

Table S1. The datasets of RNA-seq sequencing generated in this article. **Sheet 1.** Mapping statistics of RNA-seq data. **Sheet 2.** Normalized transcript expression levels of our samples. **Sheet 3.** Functional annotation statistics of transcripts identified in this

study. **Sheet 4.** GO annotation of transcripts identified in this study. **Sheet 5.** GO statistics of transcripts identified in this study. **Sheet 6.** KEGG pathway annotation of transcripts identified in this study. **Sheet 7**. KEGG pathway statistics of transcripts identified in this study. (XLS)

Table S2. Differential expression genes (DEGs) identifed in the comparison between negative group（NC） and solvent control group (SC). (XLSX)

Table S3. Expression and annotation information of acetamiprid responding DEGs based on FDR and log2 fold change. (XLSX)

Table S4. Expression and annotation information of carbaryl responding DEGs based on FDR and log2 fold change. (XLSX)

Table S5. GO classification of the carbaryl-responding genes. (XLSX)

Table S6. KEGG pathway enrichment analysis of carbaryl-responding genes. (XLSX)

Table S7. Metabolite profiling data and statistical assessments in the comparison of ACE vs. SC. (XLSX)

Table S8. Metabolite profiling data and statistical assessments in the comparison of CR vs. SC. (XLSX)

Table S9. Known metabolite identified in comparison of carbaryl (CR) or acetamiprid (ACE) vs control bees (SC). (XLSX)

Table S10. KEGG enrichment analysis of Known metabolite identified in comparison of carbaryl (CR) vs control bees (SC). (XLSX)

Table S11. Correlation analysis of DEGs and DEMs identified in the transcriptomic and metabolomic assays in the present study. (XLSX)

Table S12. KEGG enrichment analysis of DEMs and DEGs identified in comparison of carbaryl (CR) vs control bees (SC) in same pathways. (XLSX)

Table S13. Network analysis of gene-metabolite interaction under carbaryl stress generated by MetaboAnalyst database. (XLSX)

Figure S1

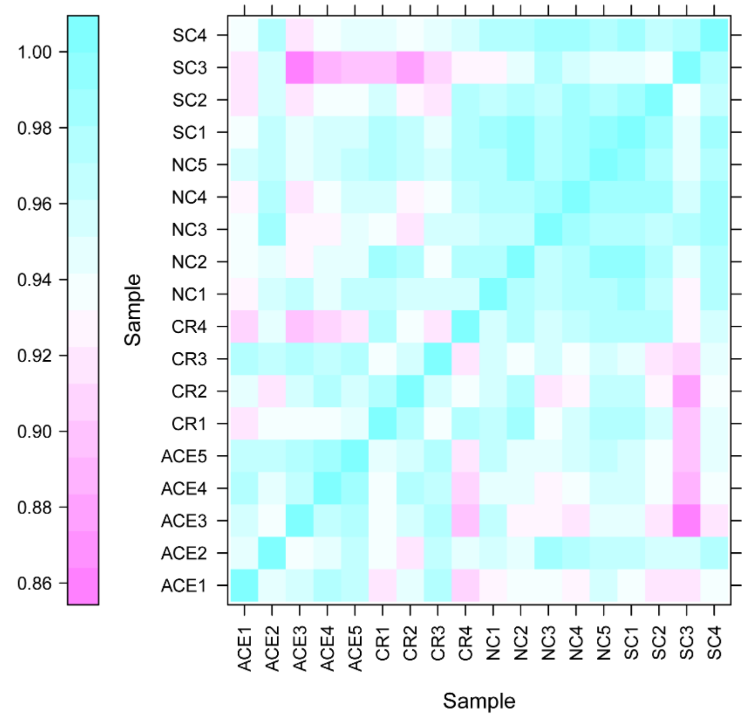

Figure S1. A Pearson correlation analysis of repetitive samples in this study.

Figure S2

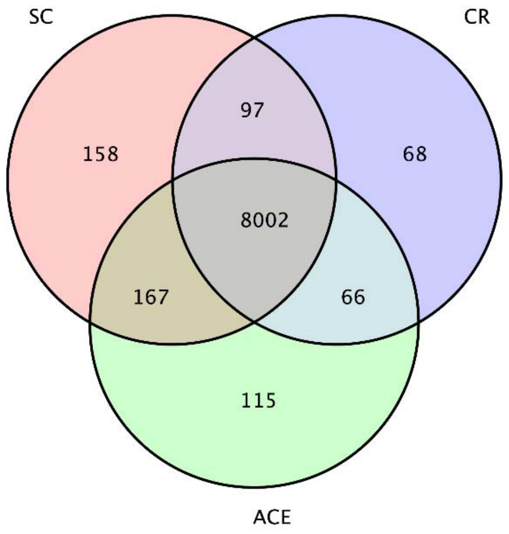

Figure. S2. Venn diagram of unigenes identified in CR (exposure to carbaryl), ACE (exposure to acetamiprid) and SC (solution control) group.

Supplementary Materials and Methods

Metabolomic profiling

Sample preparation. We sampled ten larvae from each culture plates after the treatments described above and immediately placed in liquid nitrogen and followed by long-term storage at –80°C. The samples were freeze-dried and homogenized to a fine powder at 4°C using a bead mill. Approximately 50 mg powdered samples were extracted with 1000 μL ice-cold extraction solvent (V methanol: V acetonitrile: V water = 2:2:1, containing internal standard substances 2 μg/mL) with ball mill. The mixture was centrifuged at 20 000 ×g for 15 min at 4 ˚C. Supernatant (around 850 μL) was transferred to a new 1.5 mL Eppendorf tube and processed through vacuum freeze drying before liquid chromatography separation. The polar fraction was prepared by liquid partitioning adding 300 μL extraction solvent (V acetonitrile: V water= 1:1). After centrifugation at 12000 rpm for 15 min at 4 ˚C, the supernatant was transferred into a fresh 2 mL LC/MS glass vial. Each condition was analyzed by 5 biological replicates.

Mass spectrometry. The metabolites profiling analyses was performed using an UHPLC system (1290, Agilent Technologies) coupled to TripleTOF 5600 (Q-TOF, AB Sciex). Sample analyses were performed in both positive and negative ion modes. The MS raw data (.wiff) files were converted to the mzXML format using ProteoWizard, and processed by R package XCMS (version 3.2). The preprocessing results generated a data matrix that consisted of the retention time (RT), massto-charge ratio (m/z) values, and peak intensity. R package CAMERA was used for peak annotation after XCMS data processing.

Data analysis. Metabolites were identified by comparing ion signatures in samples to purification standard entries in a metabolomics reference library (Evans et al., 2009), including retention time, molecular weight (m/z), preferred adduct, in-source fragments and associated mass spectra. Before the data analysis, QC analysis was conducted to confirm the reliability of the data. For quantitative metabolomics, data matrices with the intensity of the metabolite features from the 15 samples were uploaded to the Analyst 1.6.1 software (AB SCIEX, Ontario, Canada) for statistical analyses. Missing values for a given metabolite were assigned the observed minimum value (minimum value imputation). The data matrix was then subjected to multivariate analysis by fitting principal component analysis (PCA) and partial least squares discriminant analysis (PLS-DA) using the SIMCA-P software v.14.1 (Umetrics, Umea, Sweden). The abundance of the portion-changed compounds was then normalized using "Range scaling" according to Wilinski *et al*. (Wilinski et al., 2019). Significantly differential metabolites were screened using VIP≥1, $p<0.05$ and $|\text{fold change}| \geq 1.5$. Clustering and pathway analyses of the differentially expressed metabolites were performed and analyzed using KEGG database (http://www.genome.jp/kegg/) (Kanehisa and Goto, 2000).

Reference

Evans, A.M., DeHaven, C.D., Barrett, T., Mitchell, M., Milgram, E., 2009. Integrated, Nontargeted Ultrahigh Performance Liquid Chromatography/Electrospray Ionization Tandem Mass Spectrometry Platform for the Identification and Relative Quantification of the Small-Molecule Complement of Biological Systems. Analytical Chemistry 81, 6656-6667.

Kanehisa, M., Goto, S., 2000. KEGG: Kyoto Encyclopedia of Genes and Genomes. Nucleic Acids Research 28, 27-30.

Wilinski, D., Winzeler, J., Duren, W., Persons, J.L., Holme, K.J., Mosquera, J., Khabiri, M., Kinchen, J.M., Freddolino, P.L., Karnovsky, A., Dus, M., 2019. Rapid metabolic shifts occur during the transition between hunger and satiety in Drosophila melanogaster. Nature Communications 10.
